# Immunomodulatory Properties and Ameliorative Effects of *Pediococcus inopinatus* in an Animal Model of Inflammatory Bowel Disease

**DOI:** 10.4014/jmb.2509.09045

**Published:** 2025-11-06

**Authors:** Ho Jae Lee, Kon-Young Ji, Dong Ho Jung, Joo Young Lee, Hasun Choi, Yebin Kim, Wooje Lee, Taesoo Kim, Sungwook Chae, Sung Wook Hong

**Affiliations:** 1 Technology Innovation Research Division, World Institute of Kimchi, Gwangju 61755, Republic of Korea; 2 KM Convergence Research Division, Korea Institute of Oriental Medicine, Daejeon 34054, Republic of Korea; 3 Department of Korean Convergence Medical Science, University of Science and Technology, Daejeon 34113, Republic of Korea

**Keywords:** *Pediococcus inopinatus*, immune-enhancing effect, immunomodulatory effect

## Abstract

Inflammatory bowel disease (IBD) is a chronic gastrointestinal disorder associated with dysregulated immune responses and gut inflammation. In this study, lactic acid bacteria were isolated from various types of skate kimchi, and 100 strains were screened for their anti-inflammatory activity. WiKim0108 demonstrated potent suppression of nitric oxide production and proinflammatory cytokines (IL-1β, IL-6, TNF-α) in lipopolysaccharide (LPS)-stimulated RAW 264.7 macrophages without cytotoxic effects. Phylogenetic analysis based on 16S rRNA sequences confirmed that the strain was *Pediococcus inopinatus*, which is closely related to other species of the genus widely used in food fermentation. WiKim0108 was susceptible to 14 clinically relevant antibiotics and exhibited γ-hemolysis, indicating its safety for use in food applications. Enzymatic profiling revealed functional activities beneficial for food fermentation, including β-galactosidase activity, but β-glucuronidase activity was absent. *In vitro*, WiKim0108 enhanced immune responses, such as the proliferation of RAW 264.7 cells, production of NO and reactive oxygen species, and expression of immune-related genes. These immunomodulatory effects were validated *in vivo* through an increased population of innate and adaptive immune cells and the upregulation of immune-related genes. In a DSS-induced IBD mouse model, oral administration of WiKim0108 ameliorated clinical and histological symptoms by restoring the immune cell population and suppressing excessive expression of immune cytokine. Collectively, these findings indicate that *P. inopinatus* WiKim0108 is a safe and functional lactic acid bacterium with dual anti-inflammatory and immunomodulatory effects, highlighting its potential as a functional starter culture and bioactive food ingredient for promoting gut health.

## Introduction

Inflammatory bowel disease (IBD), which includes ulcerative colitis and Crohn’s disease, is a chronic and relapsing inflammatory disorder of the gastrointestinal tract with steadily increasing prevalence and incidence worldwide [1]. IBD has recently exhibited a marked increase in incidence, emerging as a significant global health concern. Its global prevalence is estimated at approximately 0.7%, projected to reach 0.85% by 2025, and expected to exceed 1% of the population by 2035 [2]. The pathogenesis of IBD is complex and involves an interplay between genetic predisposition, dysregulated immune responses, environmental factors, and imbalances in the gut microbiota (gut dysbiosis), which collectively contribute to chronic intestinal inflammation [1].

Current IBD treatments primarily focus on alleviating symptoms and preventing relapse using aminosalicylates, corticosteroids, immunomodulators, and biologic agents [3]. However, these therapies are associated with adverse effects, high costs, and limited long-term efficacy. Moreover, they do not effectively address gut microbiota dysbiosis, which is increasingly recognized as a fundamental contributor to disease pathogenesis [4].

Inflammation is a critical host defense mechanism; however, excessive or chronic inflammation can lead to tissue damage and the development of various chronic diseases. Recent studies have revealed that the composition and metabolic activities of the gut microbiota play key roles in maintaining immune homeostasis, increasing interest in probiotics derived from food sources as safe and mechanism-based anti-inflammatory therapeutic strategies [5]. Kimchi, a representative traditional Korean fermented food, contains diverse lactic acid bacteria (LAB) and numerous bioactive compounds produced during fermentation, including organic acids, vitamins, exopolysaccharides, and bacteriocins, which have been suggested to positively influence host immune modulation and enhance intestinal mucosal barrier function [6].

Kimchi fermentation primarily involves various LAB, including *Lactiplantibacillus*, *Leuconostoc*, *Weissella*, and *Pediococcus* spp. A few of these strains exhibit anti-inflammatory effects by modulating innate and adaptive immunity, enhancing intestinal epithelial barrier function, antagonizing pathogenic microorganisms, and promoting short-chain fatty acid production. At the molecular level, immune regulatory mechanisms, such as balancing Toll-like receptor (TLR) signaling, inhibition of NF-κB and MAPK pathways, downregulation of inducible nitric oxide synthase (iNOS) and cyclooxygenase-2 (COX-2) expression, suppression of proinflammatory cytokines, including TNF-α, interleukin (IL)-1β, and IL-6, along with upregulation of the anti-inflammatory cytokine IL-10 and induction of regulatory T cell (Treg) responses, have been reported [7]. In addition, postbiotic components, such as peptidoglycans, teichoic acids, surface proteins, and exopolysaccharides (EPS) derived from LAB cell walls, have attracted attention for their ability to modulate host immune responses through receptor binding [8].

Recent studies have highlighted probiotics as adjunctive therapeutics for IBD, which can alleviate symptoms through multiple mechanisms, including the restoration of gut microbial balance, enhancement of intestinal mucosal barrier function, and modulation of the mucosal immune response. Certain probiotic strains have demonstrated anti-inflammatory effects in animal models by suppressing the expression of inflammatory mediators and regulating immune responses, suggesting their potential to reduce the severity of IBD [9].

In this study, *P. inopinatus*, a lactic acid bacterium with immunomodulatory and ameliorative effects on IBD, was isolated and identified from the traditional fermented food Hongeo kimchi (skate kimchi). This strain exhibited no cytotoxicity, showed immune-enhancing activity, and promoted the production of nitric oxide (NO) and reactive oxygen species (ROS) in both *in vitro* and *in vivo* experiments. Furthermore, it positively modulates the expression of inflammatory cytokines and increases immune cell populations, resulting in significant improvements in animal models of IBD. These findings suggest that *P. inopinatus* has considerable potential as a functional food ingredient or pharmaceutical agent for the prevention, mitigation, and treatment of IBD via immune regulation.

## Materials and Methods

### Isolation of Strains and Culture

Commercially fermented skate kimchi (10 g) was purchased from Yeongsanpo Honggeo Co. (Republic of Korea). The sample was subjected to a 10-fold serial dilution using sterilized 0.85% (w/v) sodium chloride and subsequently plated onto de Man, Rogosa, and Sharpe (MRS) agar (Difco; BD Biosciences, USA), which is selective for LAB. After incubation at 30°C for 48 h, distinct microbial colonies were isolated based on their morphological characteristics. The isolated strains were further cultured in a minimal nutrient medium containing 1% glucose and 0.5% yeast extract at 30°C for 48 h.

### Identification of Selected Strain

The selected strain was identified using a DNeasy Tissue Kit (Qiagen, USA) according to the manufacturer’s instruction. The conserved region of the 16S ribosomal RNA (rRNA) gene was amplified using universal primer pairs (RP: (1492R) 5'-GGTTACCTTGTTACGACTT-3' and FP: (27F) 5'-AGAGTTTGATCCTGGCTCAG-3') and Takara Perfect Premix (Takara, Japan) on an Applied Biosystems thermal cycler (Thermo Fisher Scientific, USA). The conditions of polymerase chain reaction (PCR) were as follows: denaturation at 94°C for 5 min; 30 cycles of denaturation at 94°C for 45 sec, annealing at 52°C for 45 sec, extension at 72°C for 1 min, and final elongation at 72°C for 5 min. The 16S rRNA was sequenced using a Genetic Analyzer (ABI 377; Applied Biosystems, USA). The sequenced 16S rRNA gene was identified using the EZBioCloud database (www.ezbiocloud.net/eztaxon). Genetic distances and the construction of neighbor-joining, maximum-likelihood, and parsimony phylogenetic trees were performed using the MEGA software. Bootstrap analysis with 1,000 replicates was conducted for each of the three methods to assess the robustness of phylogenetic groupings [10].

### Safety Evaluation of Selected Strain

Antibiotic susceptibility of the indicator bacteria was evaluated on de MRS agar using the agar disk diffusion method. The bacterial culture was aseptically spread onto the surface of MRS plates using a sterile swab, and antibiotic disks (BD BBL, USA) were placed on the agar. The plates were left for 10–15 min at room temperature to facilitate the diffusion of antibiotics into the medium, followed by incubation at 30°C for 48 h. The tested antibiotics included: ampicillin (10 μg), cefotetan (30 μg), chloramphenicol (30 μg), ciprofloxacin (5 μg), clindamycin (2 μg), gentamicin (10 μg), doxycycline (30 μg), erythromycin (15 μg), kanamycin (30 μg), penicillin G (6 μg), streptomycin (10 μg), tetracycline (30 μg), trimethoprim-sulfamethoxazole (25 μg), and vancomycin (30 μg) [11].

The hemolytic activity of the selected strain was evaluated using blood agar plates containing 5% (v/v) sheep blood. The strain was streaked onto the surface of the medium and incubated at 30°C for 48 h. Hemolytic activity was determined by observing the presence and type of hemolysis around the colonies: β-hemolysis was indicated by a clear zone of lysis, whereas γ-hemolysis was characterized by the absence of any visible zone of lysis [12].

The enzymatic profile of the selected strain was assessed using a semi-quantitative API ZYM kit (BioMérieux, France) according to the manufacturer’s instructions. Cultures were centrifuged at 8,000 ×*g* for 15 min at 4°C, and the pellets were resuspended in sterile 0.85% NaCl solution to a concentration of 10^6^ CFU/ml. The microcapsules of the API ZYM strip were inoculated with the prepared bacterial suspension from 24-h-old broth cultures and incubated at 30°C for 4 h. Following incubation, ZYM A and ZYM B reagents were added sequentially to each couple. The strips were then visually examined under light, and the enzymatic activity was evaluated based on the intensity of color change. Enzyme activity was interpreted as negative (grades 0 and 1) or moderately to strongly positive (grades 2–5) [13].

### Cell Culture

The murine macrophage cell line RAW 264.7 (ATCC, USA) was cultured in Dulbecco’s Modified Eagle’s Medium (Gibco, Thermo Fisher Scientific) supplemented with 10% fetal bovine serum (FBS; Gibco) and 1% antibiotics (100 units/ml penicillin and 100 μg/ml streptomycin; Gibco). The cells, confirmed to be free of *Mycoplasma* contamination, were maintained at 37°C in a humidified atmosphere of 5% CO_2_ and used for experiments up to passage 18.

### Animals

Animal experiments were approved by the Institutional Animal Care and Use Committee of the Korea Institute of Oriental Medicine (approval number: 18-042). C57BL/6 mice (6 weeks old, male) were purchased from Japan SLC, Inc. (Japan), and all mice were maintained in specific pathogen-free conditions and controlled environment (temperature: 24 ± 2°C, humidity: 50% ± 5%, light/dark cycle: 12 h) with free access to food and water.

### Dextran Sodium Sulfate (DSS)-induced IBD Mice Model

Mice were randomly separated into four groups (wild-type [WT] control, DSS-induced control, DSS-induced 5-aminosalicylic acid (5-ASA) positive control, and DSS-induced WK0108; *n* = 6 each) and maintained for 1 week for adaptation. After the adaptation period, the mice in the four groups were administered 100 μl of PBS or 100 mg/kg/100 μl of 5-ASA (Sigma-Aldrich, USA), 1 × 10^8^ CFU/100 μl of the WiKim0108 strain by oral gavage daily during the experimental period. After 6 days, DSS-induced mice were administered drinking water containing 2% w/v DSS (36,000-50,000 kDa; MP Biomedical, USA). The mice were euthanized after anesthesia for further investigations.

### Analysis of Cell Proliferation and NO Production

RAW 264.7 cells were seeded into 96-well plates at a density of 1 × 10^5^ cells/ml and incubated for 24 h. The culture medium was replaced with fresh medium containing 1 μg/ml LPS (Sigma Aldrich) or the indicated concentration of the culture supernatant of (heat-killed) WiKim0108 strain, followed by incubation for 24 h. The cell proliferation was measured using an MTS solution kit (CellTiter 96 AQueous One Solution Cell Proliferation Assay; Promega, USA) according to the manufacturer’s protocol. NO production in the cell culture supernatants was measured using the Griess reagent system (Promega) according to the manufacturer’s instructions.

### Measurement of ROS Production

RAW 264.7 cells (1 × 10^5^ cells/ml) were cultured in 12-well plates and incubated for 24 h. The cells were stimulated with 1 μg/ml of LPS or the indicated concentration of the culture supernatant of (heat-killed) WiKim0108 strain for 24 h. The cells were washed with ice-cold PBS (Gibco) containing 1% FBS and stained with CellROX Deep Red Reagent (Thermo Fisher Scientific) according to the manufacturer’s instructions. The population of ROS-positive cells was analyzed using an LSRFortessa X-20 flow cytometer (BD Bioscience, USA) and FlowJo v. 10 software (FlowJo, USA).

### Real-Time PCR Analysis of Inflammatory Cytokines

Total RNA was isolated and purified from RAW 264.7 cells or the spleen of DSS-induced IBD mice using the RNeasy Mini Kit (Qiagen, USA) according to the manufacturer’s protocol. Purified RNA was used to synthesize cDNA using TaqMan reverse transcription reagent (Applied Biosystems, USA). Primers for β-actin, iNOS, IL-1β, IL-6, TNF-α, and cyclooxygenase 2 (COX-2) were purchased from TaqMan Gene Expression Assay (Applied Biosystems). Gene expression levels were quantified using the ABI QuantStudio 6 Flex RT-PCR system and TaqMan Universal PCR Master Mix (Applied Biosystems) following the manufacturer’s instructions. mRNA levels were normalized to those of β-actin and calculated using the ΔΔCt method.

### Analysis of the Immune Cell Population

Upon sacrifice, the spleens were excised and passed through a 40 μm cell strainer (Falcon, USA). Red blood cells were removed using RBC lysis buffer (BioLegend, USA) according to the manufacturer’s protocol. The cells were resuspended in fluorescein-activated cell sorter (FACS) staining buffer (BD Biosciences), and 1 × 10^6^ cells were stained with the indicated antibodies for 20 min on ice. The FACS antibodies against CD4-FITC and CD8-antigen-presenting cells (APC), CD45R/B220- FITC, NK-1.1-APC, CD69-PE and CD11b-APC and CD11c-FITC were purchased from BD Biosciences. The population of immune cells was analyzed using an LSRFortessa X-20 flow cytometer (BD Bioscience) and FlowJo v. 10 software (FlowJo).

### Evaluation of DSS-Induced IBD Symptoms

DSS-induced IBD symptoms were evaluated using previously described protocols with minor modifications [14, 15]. Briefly, weight loss, stool consistency, and visible blood in the rectum and feces were recorded daily. The disease activity index (DAI) was calculated by scoring the average score for each symptom (Table 1). Colon length was measured between the ileocecal junction and rectum after the mice were sacrificed. For histological analysis, the colon tissues were stained with hematoxylin and eosin (H&E; Sigma--Aldrich), and the scores were calculated as shown in Table 2.

### Statistical Analysis

The *in vitro* data are shown as mean ± standard deviation, and the *in vivo* data are reported as the mean ± standard error of the mean. Significance was calculated using one-way analysis of variance, followed by Tukey’s multiple comparison test using GraphPad Prism version 9 (GraphPad, USA). Statistical significance was set at *p*<0.05.

## Results and Discussion

### Isolation and Identification of LAB with Anti-Inflammatory Activity

A total of 100 LAB isolated from various types of kimchi belonging to the genera *Lactobacillus*, *Leuconostoc*, *Pediococcus*, and *Weissella* were screened for anti-inflammatory activity in their culture supernatants. Based on the preliminary screening, 10 strains exhibiting strong activity were selected for further evaluation. The anti-inflammatory potential of these strains was assessed in LPS-stimulated RAW 264.7 macrophages. Among the tested strains, WiKim0108 significantly inhibited NO production and markedly reduced the levels of proinflammatory cytokines, including TNF-α, IL-1β, and IL-6, compared to those in the LPS-treated control. In addition, WiKim0108 treatment enhanced macrophage viability without causing cytotoxicity. Repeated experiments confirmed the reproducibility of these findings, and WiKim0108 consistently exhibited the strongest anti-inflammatory activity among the selected strains (data not shown).

**Fig. 1:**
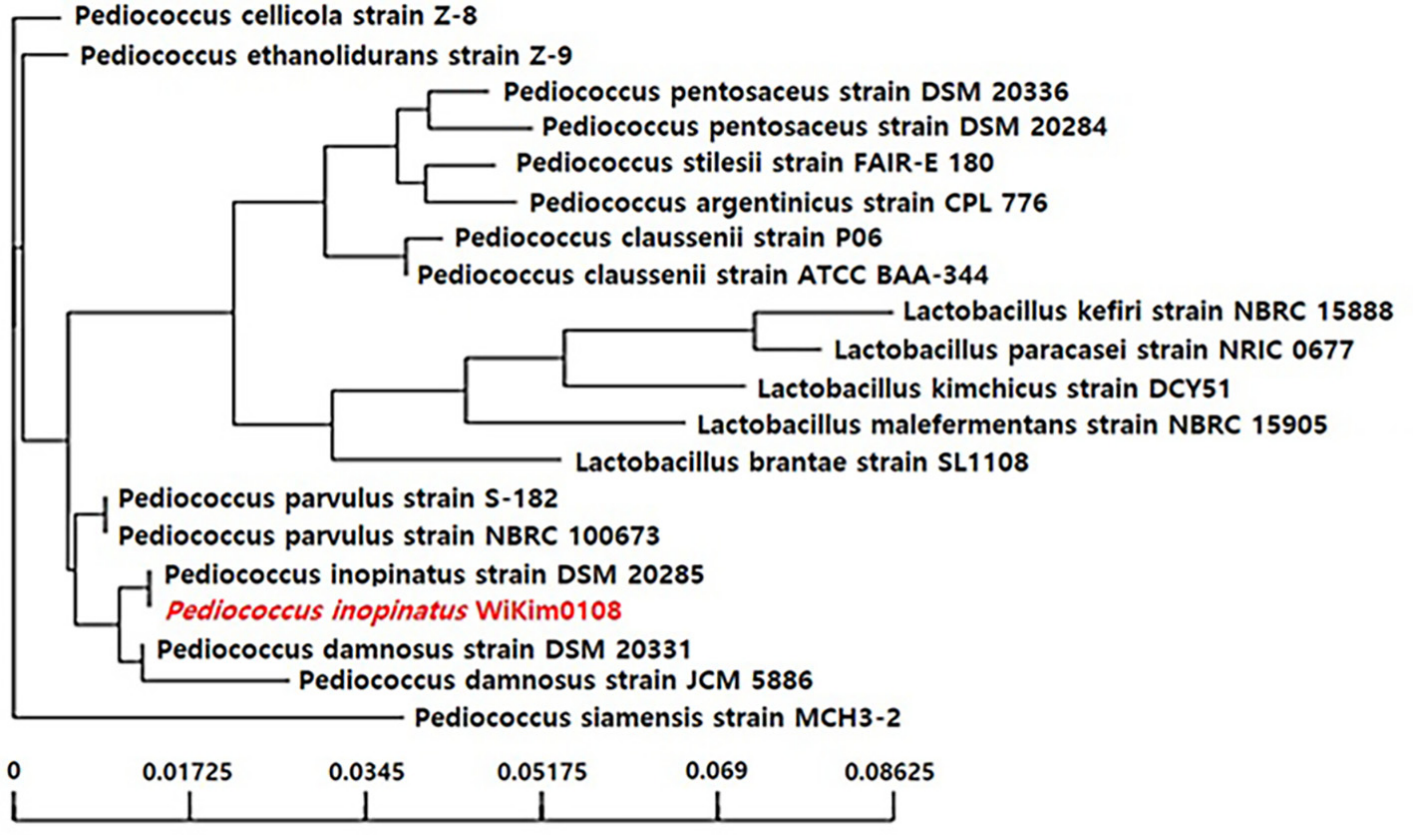
Phylogenetic relationships of *Pediocwoccus inopinatus* WiKim0108 based on 16S rRNA gene sequence. Phylogenetic tree constructed using the neighbor-joining method based on 16S rRNA gene sequences. Bootstrap values (1,000 replicates) are indicated at the nodes to assess the reliability of the branching. The scale bar represents the number of substitutions per site, reflecting the evolutionary distance among strains.

These results indicate that WiKim0108 can effectively modulate the production of inflammatory mediators while maintaining cell viability, suggesting its potential as a safe and effective anti-inflammatory agent. Therefore, 16S rRNA gene sequencing was performed to identify the bacterial strains. The strain WiKim0108, isolated from a skate kimchi, showed 97% sequence similarity to the reference strain *P. inopinatus* BT and was therefore designated as *P. inopinatus* WiKim0108 (Fig. 1).

The species *P. inopinatus* was first described by Back (1978) in a taxonomic study, in which it was characterized as a novel subspecies associated with spoilage in beer and was formally named *P. inopinatus* [16]. *P. inopinatus* was subsequently isolated and identified from kimchi by Lim *et al*. (1989) [17], who re-evaluated gram-positive bacterial populations inhabiting this traditional Korean fermented food. These findings highlight that *P. inopinatus* is a kimchi-derived lactic acid bacterium with a long history of safe dietary exposure through traditional consumption methods. *P. inopinatus* belongs to the genus *Pediococcus* within the family *Lactobacillaceae*, and along with *P. acidilactici*, *P. pentosaceus*, and *P. damnosus*, it is recognized as a representative lactic acid bacterium that is widely used in food fermentation and considered safe for consumption. The phylogenetic analysis based on 16S rRNA sequences conducted in this study reflects such intrageneric relatedness and supports the close genetic and taxonomic relationships of this species with other members of the genus *Pediococcus* [18]. Members of the genus *Pediococcus* have been extensively used as starter cultures or beneficial starter strains in various food fermentation processes, including those of dairy products, meat, kimchi, and cheese. They are known to produce bacteriocins, such as pediocins, which contribute significantly to food preservation and antimicrobial activity [19].

### Antibiotic Susceptibility

The antibiotic susceptibility of *P. inopinatus* WiKim0108 was evaluated using the agar disk diffusion method on MRS agar. *P. inopinatus* WiKim0108 strain was susceptible to β-lactams and several protein synthesis inhibitors. Based on the mean inhibition zone diameters, ampicillin, penicillin, doxycycline, clindamycin, chloramphenicol, erythromycin, and tetracycline were classified as susceptible according to the Clinical and Laboratory Standards Institute (CLSI) criteria. In contrast, vancomycin, gentamicin, streptomycin, ciprofloxacin, and trimethoprim/sulfamethoxazole produced small inhibition zones and were considered resistant (Table 3). Compared with the reference strains reported in the European Food Safety Authority (EFSA) guidelines, the susceptibility profile of WiKim0108 broadly followed the patterns commonly observed within the genus *Pediococcus*. The WiKim0108 strain was susceptible to clinically relevant macrolides (erythromycin), a lincosamide (clindamycin), and tetracyclines, whereas the large inhibition zones against β-lactams (ampicillin and penicillin; >39 mm) indicated a high sensitivity to cell wall-targeting antibiotics [20]. These findings indicate that the antibiotic susceptibility profile of *P. inopinatus* WiKim0108 follows the patterns reported for the genus *Pediococcus*, suggesting its potential suitability as a safe probiotic. The pronounced susceptibility to β-lactams and clinically important macrolides, lincosamides, and tetracyclines underscores their favorable safety profiles. Nevertheless, further confirmation through MIC-based assays and additional evaluations of resistance and virulence determinants are warranted to establish compliance with the regulatory standards for food and functional applications.

### Hemolytic Activity

**Fig. 2:**
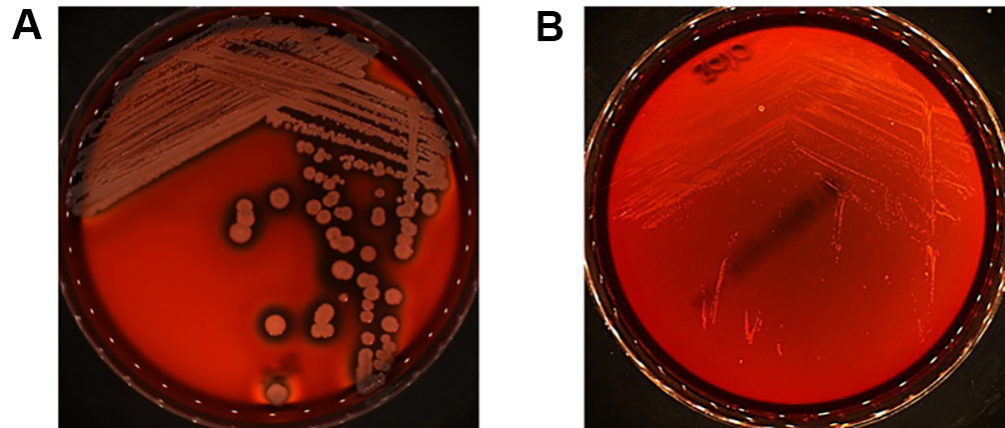
Hemolytic activity of *Pediococcus inopinatus* WiKim0108 on sheep blood agar. (**A**) *S. aureus* ATCC 25923; (**B**) *P. inopinatus* WiKim0108. Hemolytic activity of the strain on 5% sheep blood agar. Colonies were incubated at 30°C for 48 h, and hemolysis was evaluated based on the presence or absence of clear (β-hemolysis) or absent (γ-hemolysis) zones around the colonies.

The hemolytic activity of *P. inopinatus* WiKim0108 was assessed using blood agar plates containing 5% (v/v) sheep blood. No hemolytic activity was observed in WiKim0108, as evidenced by the absence of clear or discolored zones around the colonies (Fig. 2). This result indicates that WiKim0108 exhibits γ-hemolysis and does not possess hemolytic activity, supporting its safety. In previous studies, *P. inopinatus* strains isolated from kimchi exhibited γ-hemolysis, indicating their nonpathogenicity [19, 23]. This provides important criteria for evaluating the safety of these strains as potential food ingredients in the future.

### Enzymatic Activities

The enzymatic activities of *P. inopinatus* WiKim0108 were analyzed using the API ZYM system, a rapid semi-quantitative assay that detects 19 enzymatic reactions. WiKim0108 exhibited considerable activity of leucine arylamidase, valine arylamidase, α-glucosidase, and β-glucosidase (Table 4). β-Glucuronidase, an enzyme produced by intestinal bacteria, is secreted as part of microbial metabolism. When harmful compounds enter the body, they are detoxified in the liver via conjugation with glucuronic acid; however, β-glucuronidase can deconjugate these glucuronides after they are excreted into the intestinal lumen with bile, thereby regenerating the parent toxins and restoring their potential toxicity [24]. β-glucuronidase activity was not detected in WiKim0108, similar to other *P. inopinatus* strains, indicating a low potential for producing carcinogenic metabolites [25]. These enzymatic characteristics suggest that *P. inopinatus* WiKim0108 possesses functional properties that are beneficial for food fermentation and does not exhibit harmful enzymatic activities. These results support the suitability as a safe and functional starter culture for fermented food production [26].

### Immunomodulatory Effect of *P. inopinatus* in RAW 264.7 Cells

**Fig. 3:**
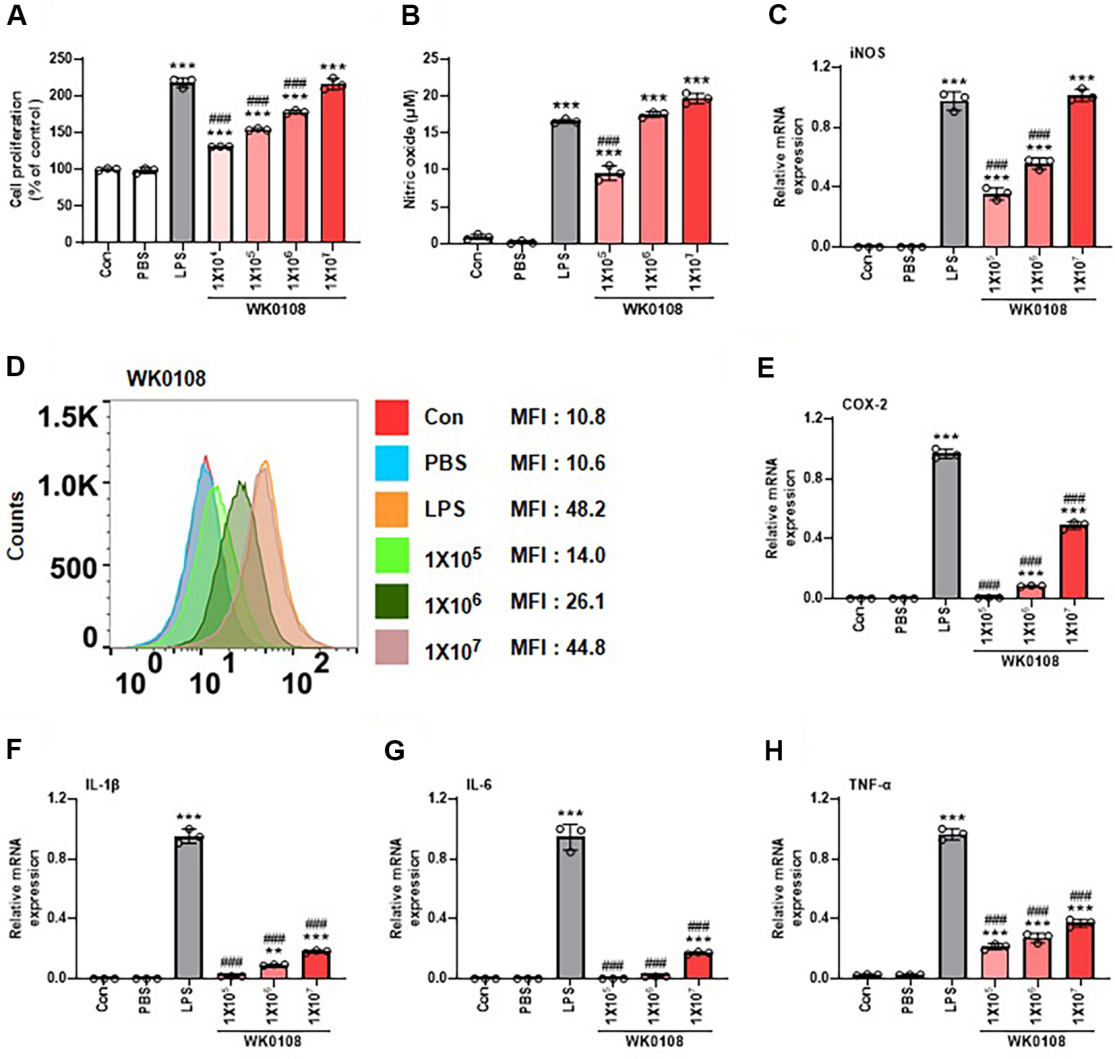
Enhancement of immune responses in RAW 264.7 cells by treatment with *Pediococcus inopinatus* WiKim0108. RAW 264.7 cells were treated with WiKim0108 for 24 h. (**A**) Changes in cell proliferation by WiKim0108 were analyzed using the MTS assay. The production of nitric oxide (NO) (**B**) and reactive oxygen species (ROS) (**D**) was measured using the Griess reagent system and CellROX Deep Red reagent, respectively. Gene expression of inducible nitric oxide synthase (iNOS) (**C**), cyclooxygenase-2 (COX-2) (**E**), IL-1β (**F**), IL-6 (**G**), and TNF-α (**H**) was analyzed using RT-PCR. Data are reported as mean ± standard deviation (SD). **p < 0.01, ***p < 0.001 vs. control (Con). ^###^*p* < 0.001 vs. lipopolysaccharide control (LPS). WK0108, *Pediococcus inopinatus* WiKim0108.

To investigate the ability of *P. inopinatus* WiKim0108 (WK0108) to modulate immune responses, RAW 264.7 cells were treated with different concentrations of the strain, and parameters including cell proliferation, NO and ROS production, and cytokine expression were analyzed. Treatment with WiKim0108 induced a dose-dependent increase in the proliferation of RAW 264.7 cells compared to the control group, although the effect was lower than that of LPS, except at 1 × 10^7^ CFU/ml, which showed comparable proliferation (Fig. 3A). Similarly, WiKim0108 stimulated NO and ROS production compared to that in the control (Fig. 3B and 3D). Although the effect on NO production at 1 × 10^5^ CFU/ml was lower than that of LPS, this difference was not observed at higher concentrations (1 × 10^6^ and 1 × 10^7^ CFU/ml) (Fig. 3B). The WiKim0108-induced increase in ROS was lower than that induced by LPS, except at a concentration of 1 × 10^7^ CFU/ml (Fig. 3D).

Gene expression analysis revealed that WiKim0108 upregulated NO- and ROS-related genes, including iNOS and COX-2, in a dose-dependent manner (Fig. 3C and 3E), consistent with the observed increase in NO and ROS levels. Except for iNOS expression at 1 × 10^7^ CFU/ml, the expression levels of iNOS and COX-2 were lower than those induced by LPS alone. In addition, WiKim0108 treatment increased the expression of immune-related cytokines, such as IL-1β, IL-6, and TNF-α, compared to the control, although these levels were lower than those in the LPS-treated group (Fig. 3F-3H). Collectively, these results indicate that WiKim0108 exerts immunomodulatory effects on RAW 264.7 macrophages by promoting cell proliferation, NO and ROS production, and cytokine expression. The magnitude of these responses was lower than that induced by LPS, suggesting that WiKim0108 enhances host immune activity without eliciting excessive inflammation, which may be advantageous for its clinical or functional applications.

These findings are consistent with previous reports demonstrating the strain-specific effects of *Pediococcus* species on the immune response. Woo *et al*. (2024) reported that *Pediococcus acidilactici* strains attenuated LPS-induced inflammation in RAW 264.7 cells by downregulating iNOS, COX-2, and proinflammatory cytokines [21]. Conversely, other studies have reported the immunostimulatory activities of *Pediococcus* spp., including elevated NO and cytokine production, which were attributed to fermentation-derived metabolites, cell-associated fractions, or extracellular polysaccharides (EPS) [27, 28]. Mechanistically, LAB, including *Pediococcus* spp., influence macrophage function via multiple pathways. Cell wall components, such as peptidoglycan and teichoic acids, activate TLRs, whereas fermentation-derived metabolites or EPS modulate NF-κB and MAPK signaling. In addition, bacteriocins and small molecules may indirectly shape the gut microbiota, contributing to secondary immune modulation [28, 29]. In the case of WiKim0108, the observed upregulation of iNOS, COX-2, and proinflammatory cytokine mRNAs, although lower than that of LPS, reflected moderate immune activation, supporting host defense while minimizing the risk of hyperinflammation [30].

### Immunomodulatory Effect of *P. inopinatus* in an *In Vivo* Model

**Fig. 4:**
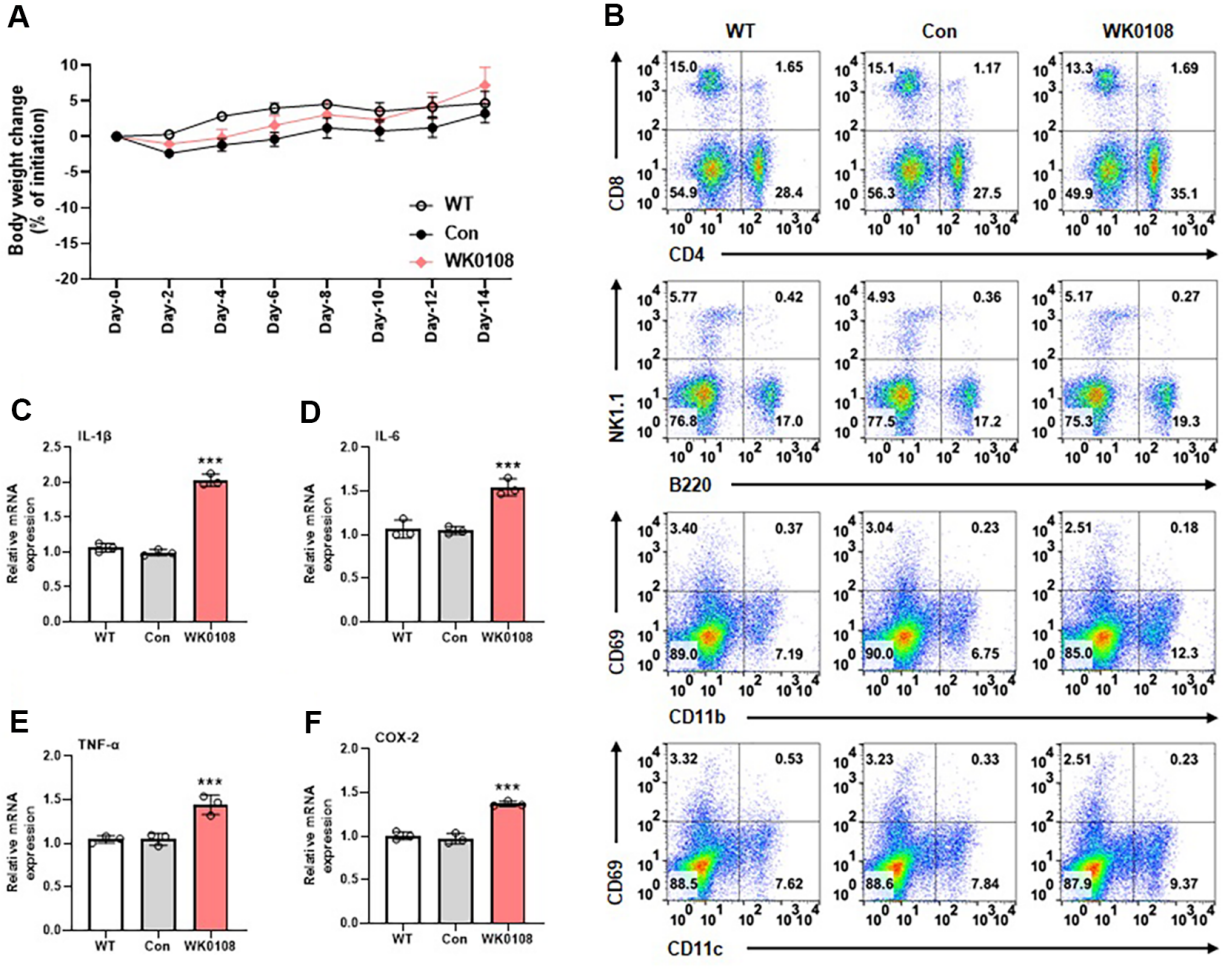
Immunomodulatory effects of *Pediococcus inopinatus* WiKim0108 on splenic immune cell populations in C57BL/6 mice. WiKim0108 was administered to C57BL/6 mice for 2 weeks. (**A**) Body weight changes were measured every other day for 2 weeks. (**B**) Immune cell populations in splenocytes were analyzed using flow cytometry. Gene expression of IL-1β (**C**), IL-6 (**D**), TNF-α (**E**), and COX-2 (F) was evaluated using RT-PCR. Data are reported as mean ± SD. ***p < 0.001 vs. control (Con). WK0108, *Pediococcus inopinatus* WiKim0108.

To evaluate the immunomodulatory effects of *P. inopinatus* WiKim0108 (WK0108) in an *in vivo* model, C57BL/ 6 mice were orally administered WiKim0108 for 14 days. First, to assess the potential toxicity of WiKim0108, changes in body weight were monitored throughout the experimental period. No significant differences were observed in body weight changes between the control and WiKim0108-administered groups, suggesting that WiKim0108 was not toxic (Fig. 4A). Immune cell populations were analyzed in mouse spleen. Compared to the control group, WiKim0108 administration increased the population of CD4 T cells (CD4^+^), B cells (B220^+^), macrophages (CD11b^+^), and dendritic cells (CD11c^+^) (Fig. 4B). We investigated the expression of immune-related genes in the spleens. Consistent with the *in vitro* results, the expression of immune-related genes, including IL-1β, IL-6, TNF-α, and COX-2, was upregulated in the WiKim0108-administered group compared to that in the control group (Fig. 4C).

These findings demonstrate that *P. inopinatus* WiKim0108 exhibits *in vivo* immunomodulatory activity, as evidenced by the increased immune cell populations and upregulation of immune-related gene expression. Oral administration of WiKim0108 did not affect the body weight, indicating the absence of overt toxicity. WiKim0108 treatment enhanced both innate and adaptive immune responses, as reflected by the elevated population of APCs, including macrophages and dendritic cells, which play pivotal roles in the initiation of adaptive immunity. This immunostimulatory effect was further supported by the increased expression of cytokines, such as IL-1β, IL-6, TNF-α, and COX-2.

These *in vivo* observations are consistent with those of previous studies on other *Pediococcus* strains, which reported strain-dependent immunomodulatory effects in animal models [31, 32]. *P. pentosaceus* strains mitigate inflammation in atopic dermatitis models via IL-10 induction and Treg cell promotion, whereas *P. acidilactici* strains improve pathology and immune balance in enteritis and allergy models, suggesting that *Pediococcus* species can modulate both innate and adaptive immunity. WiKim0108 enhanced innate immunity, as evidenced by increased splenic APC populations, which likely facilitated antigen presentation and subsequent T/B cell-mediated adaptive responses. These effects may be mediated by probiotic surface components (*e.g.*, peptidoglycan and teichoic acids) and secreted metabolites, such as EPS, which engage PRRs and activate NF-κB/MAPK signaling. The observed upregulation of iNOS, COX-2, and proinflammatory cytokines likely reflects these pathways, indicating that WiKim0108 enhances immunity without causing excessive inflammation. *In vivo* studies on *Pediococcus* strains have shown heterogeneity; some strains suppress proinflammatory cytokines, whereas others, such as WiKim0108, stimulate immune cell proliferation and cytokine production, with effects dependent on the strain, dosage, administration duration, animal model, and form (whole cells vs. cell-free supernatant).

### Preventive Effect of *P. inopinatus* in a DSS-Induced IBD Mouse Model

**Fig. 5:**
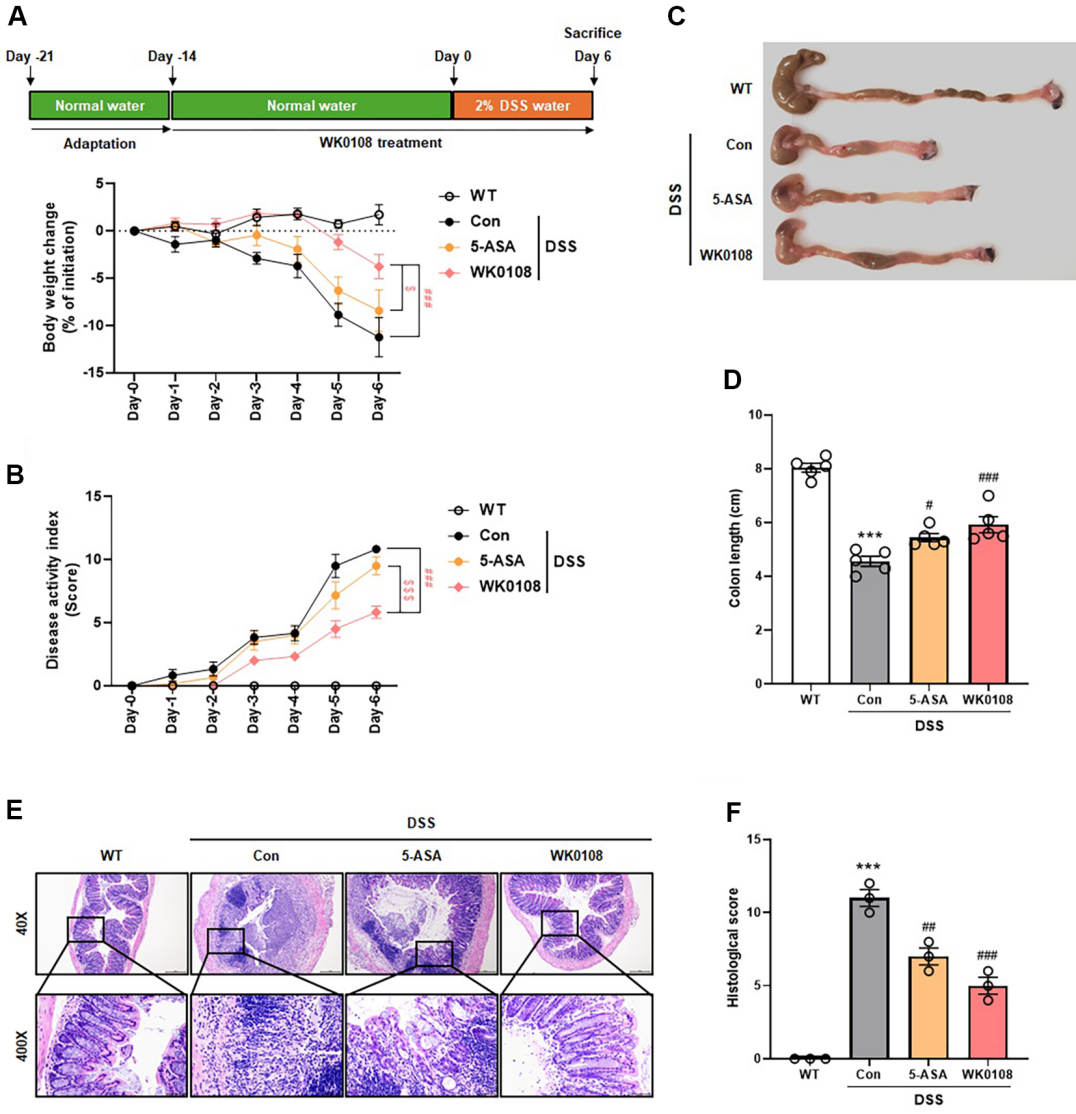
Ameliorating effects of *Pediococcus inopinatus* WiKim0108 on clinical and histological signs of DSSinduced IBD. C57BL/6 mice were treated with 2% dextran sodium sulfate (DSS) for 6 days. Body weight changes (**A**) and disease activity index (DAI) scores (**B**) were measured on alternate days. Images of the colon tissue (**C**) and colon length (**D**) were analyzed after sacrificing mice with DSS-induced inflammatory bowel disease (IBD). Microscopic images (**E**) and histological scores (**F**) were assessed and calculated after staining the colon tissues with hematoxylin and eosin. The colon tissue images were magnified 40× and 400×. Data are reported as mean ± standard error of the mean (SEM). ***p < 0.001 vs. WT. ^#^*p* < 0.05, ^##^*p* < 0.01, ^###^*p* < 0.001 vs. DSS-induced control (Con). ^$^*p* < 0.05, ^$$$^*p* < 0.001 vs. 5-ASA positive control (5-ASA). WK0108, *Pediococcus inopinatus* WiKim0108.

**Fig. 6:**
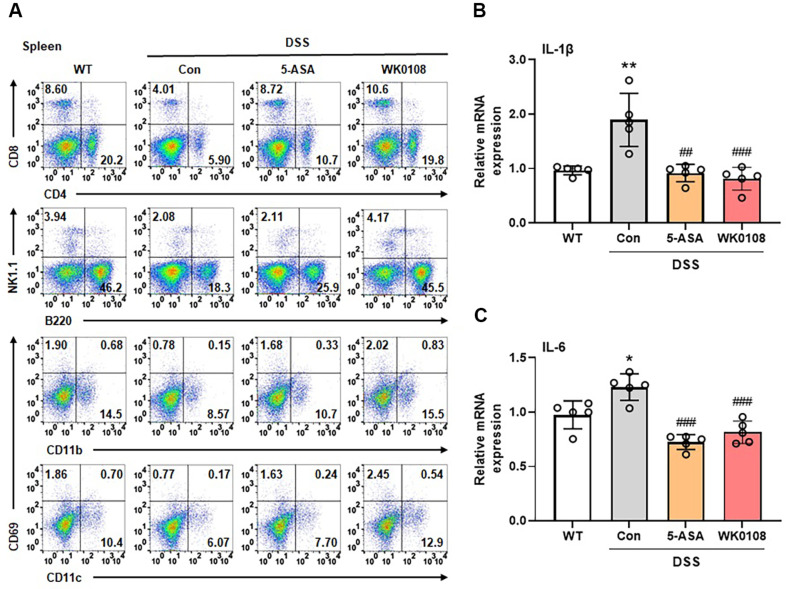
Restorative effects of *Pediococcus inopinatus* WiKim0108 on immune cell population and inflammatory cytokine levels in the spleen of DSS-induced IBD mice. (**A**) Splenocytes were isolated from DSS-induced IBD mice, and the frequency of immune cell populations was investigated using flow cytometry. Gene expression of IL-1β (**B**) and IL-6 (**C**) was analyzed using RT-PCR. Data are reported as mean ± SEM. *p < 0.05, **p < 0.01 vs. wild-type (WT). ^##^*p* < 0.01, ^###^*p* < 0.001 vs. DSS-induced control (Con). WK0108, *Pediococcus inopinatus* WiKim0108.

WiKim0108 (WK0108) exhibited immunomodulatory effects both *in vitro* and *in vivo*. Based on our findings, we evaluated the potential of WiKim0108 for the treatment of DSS-induced IBD. Although the DSS-induced control group exhibited body weight loss compared to the WT group, this phenomenon was significantly alleviated by WiKim0108 administration (Fig. 5A). Consistent with body weight loss, DAI scores continuously increased in the DSS-induced control group compared to the WT group during the experimental period, whereas this increase was significantly attenuated in the WiKim0108-administered group (Fig. 5B). Significant colon shortening was observed in the DSS-induced control group compared to the WT group; however, this trend was ameliorated by WiKim0108 administration (Fig. 5C and 5D). Histological analysis revealed severe epithelial injury in the colon tissues and significantly increased histological scores in the DSS-induced control group compared to those in the WT group, whereas these histological changes were mitigated by the administration of WiKim0108 (Fig. 5E and 5F). Analysis of immune cell populations in the spleen showed reduced frequencies of CD T cells (CD8^+^), CD4 T cells (CD4^+^), NK cells (NK1.1^+^), B cells (B220^+^), macrophages (CD11b^+^), and dendritic cells (CD11c^+^) in the DSS-induced control group; however, this reduction was reversed in the WiKim0108-administered group (Fig. 6A). Furthermore, the excessive expression of immune cytokines, such as *IL-1β* and *IL-6*, induced by DSS, was significantly suppressed by WiKim0108 administration (Fig. 6B and 6C).

Previous studies have reported similar *in vivo* immunomodulatory effects of various *Pediococcus* strains. *P. acidilactici* protects intestinal barrier function in DSS-induced colitis models by preventing epithelial cell damage and maintaining the expression of tight junction proteins. In addition, it reduces the expression of proinflammatory cytokines, such as TNF-α and IL-1β, while promoting the production of the anti-inflammatory cytokine IL-10, contributing to the restoration of immune homeostasis [33]. *P. pentosaceus* exert anti-inflammatory effects by modulating gut microbiota. Administration of *P. pentosaceus* to DSS-induced colitis models increases the abundance of beneficial bacteria, such as *Bifidobacterium* and *Lactobacillus*, and decreases the abundance of potentially pathogenic Proteobacteria, improving the intestinal microbial environment [34]. In contrast, WiKim0108 exhibited distinctive effects, suppressing intestinal tissue damage and inflammation and promoting the recovery of diverse immune cell populations. WiKim0108 normalized the composition of splenic immune cells, indicating that its immunomodulatory activity extends to systemic immune regulation.

These results indicate that the immunomodulatory effects of WiKim0108, previously observed *in vitro* and *in vivo*, can be effectively harnessed to prevent DSS-induced IBD. Oral administration of WiKim0108 alleviated both clinical and histopathological manifestations of DSS-induced IBD by restoring the immune cell populations and suppressing the overexpression of proinflammatory cytokines. These protective effects are likely due to the ability of WiKim0108 to maintain immune homeostasis by modulating the host immune response. These findings suggest that WiKim0108 is a promising candidate for the development of functional probiotics or biotherapeutic agents for IBD prevention and management.

## Conclusion

This study demonstrates that *P. inopinatus* WiKim0108, isolated from skate kimchi, is a safe and functional lactic acid bacterium with immunomodulatory properties that ameliorates IBD. The strain showed no hemolytic activity, was susceptible to clinically relevant antibiotics, and exhibited beneficial enzymatic activities while lacking harmful enzymes such as β-glucuronidase. WiKim0108 promoted immunomodulatory activities both *in vitro* and *in vivo*, including enhancement of immune cell proliferation, modulation of NO and ROS production, and upregulation of immune-related genes. These immunomodulatory effects effectively attenuated the clinical and histological symptoms of DSS-induced IBD. Collectively, these findings highlight the potential of WiKim0108 as a functional lactic acid bacterium or starter culture capable of promoting gut health by modulating immune responses and attenuating intestinal inflammation, supporting its application in health-promoting fermented food products.

## Figures and Tables

**Table 1:** Scoring system for DAI in the DSS-induced colitis.

Score	Weight loss	Stool consistency	Visible blood in rectal and feces
0	No weight loss	Well form pellets	No bleeding
1	1–5%		
2	5–10%	Loose stool	Slight bleeding
3	10–15%		
4	Over 15%	Diarrhea	Gross bleeding

DAI, disease activity index; DSS, Dextran sodium sulfate

**Table 2:** Scoring system for histological analysis in DSS-induced colitis.

Score	Histological feature			
	Loss of epithelium	Crypt damage	Depletion of goblet cells	Infiltration of inflammatory cells
0	None	None	None	None
1	0–5%	0–10%	Mild	Mild
2	5–10%	10–20%	Moderate	Moderate
3	Over 10%	Over 20%	Severe	Severe

**Table 3:** Antibiotic susceptibility of *Pediococcus inopinatus* Wikim0108 evaluated based on CLSI criteria and comparison with other *Pediococcus* spp. evaluated based on EFSA criteria.

NO	Antibiotics	*Pediococcus inopinatus* WiKim0108	*Pediococcus acidilactici* SY23 [21]	*Pediococcus pentosaceus* MZF16 [22]
1	Ampicillin	S^c^	S^b^	S
2	Vancomycin	R	-	R
3	Gentamicin	R	R	R
4	Streptomycin	R	R	S
5	Erythromycin	S	S	S
6	Clindamycin	S	S	-
7	Tetracycline	S	S	S
8	Chloramphenicol	S	S	R
9	Ciprofloxacin	R	-	-
10	Doxycycline	S	-	-
11	Penicillin	S	-	-
12	Trimethoprim/Sulfamethoxazole	R	-	-

^a^CLSI, Clinical and Laboratory Standards Institute

^b^EFSA, European Food Safety Authority

^c^R, resistant; S, susceptible; -, not determined

**Table 4:** Enzymatic activity profile of *Pediococcus inopinatus* WiKim0108 determined by the API ZYM system.

Enzyme	Enzyme activity	Enzyme	Enzyme activity
Control	0^a^	Acid phosphatase	0
Alkaline phosphatase	0	Naphthol-AS-BI-phosphohydrolase	2
Esterase	0	α-Galactosidase	0
Esterase lipase	0	β-Galactosidase	0
Lipase	0	β-Glucuronidase	0
Leucine arylamidase	5	α-Glucosidase	4
Valine arylamidase	5	β-Glucosidase	4
Cystine arylamidase	0	N-Acetyl-β-glucosaminidase	0
Trypsin	0	α-Mannosidase	0
α-Chymotrypsin	0	α-Fucosidase	0

^a^Color intensity score. 0, 0 nM; 1, 5 nM; 2, 10 nM; 3, 20 nM; 4, 30 nM; 5, ≥40 nM

The enzymatic activity profile of the selected strain was determined using API ZYM system. Activities were evaluated after 4 h of incubation at 30°C and expressed as negative (grades 0–1) or positive (grades 2–5).
